# Video-assisted thoracoscopic left lower lobectomy in a patient with lung cancer and a right aortic arch

**DOI:** 10.1186/1749-8090-7-120

**Published:** 2012-11-13

**Authors:** Hideyuki Wada, Yasuhiro Hida, Kichizo Kaga, Ryunosuke Hase, Kazuto Ohtaka, Jun Muto, Nakada-Kubota Reiko, Satoshi Hirano, Yoshiro Matsui

**Affiliations:** 1 Department of Cardiovascular and Thoracic Surgery, Hokkaido University Graduate School of Medicine, North 15, West 7, Kita-ku, Sapporo, 060-8638, Japan; 2 Department of Gastroenterology Surgery II, Hokkaido University Graduate School of Medicine, Sapporo, Japan

## Abstract

A right aortic arch is a rare congenital anomaly, with a reported incidence of around 0.1%. A patient with a right aortic arch underwent video-assisted thoracic surgery left lower lobectomy and mediastinal lymph node dissection for squamous cell carcinoma. There was no aortic arch or descending aorta in the left thoracic cavity, but the esophagus. There was no anomaly in the location or branching of the pulmonary vessels, the bronchi, and the lobulation of the lungs. The vagus nerve was found at the level of the left pulmonary artery. The arterial ligament was found between the left subclavian artery and the left pulmonary artery. The recurrent laryngeal nerve was recurrent around the left subclavian artery. A Kommerell diverticulum was found at the origin of the left subclavian artery. The patient experienced no complications. We conclude that video-assisted thoracoscopic lobectomy with mediastinal dissection is feasible for treating lung cancer with a right aortic arch.

## Background

A right aortic arch is a relatively rare congenital anomaly, with a reported incidence of around 0.1% [1]. In cases of pulmonary resection for the left lung cancer with a right aortic arch, it is important to locate the vasculature and nerves, especially the recurrent laryngeal nerve (RLN), to avoid injury. We report a case in which we could identify the RLN under video-assisted thoracic surgery (VATS). It was possible to perform lobectomy with mediastinal lymph node dissection without any complications.

## Case presentation

**Figure 1:**
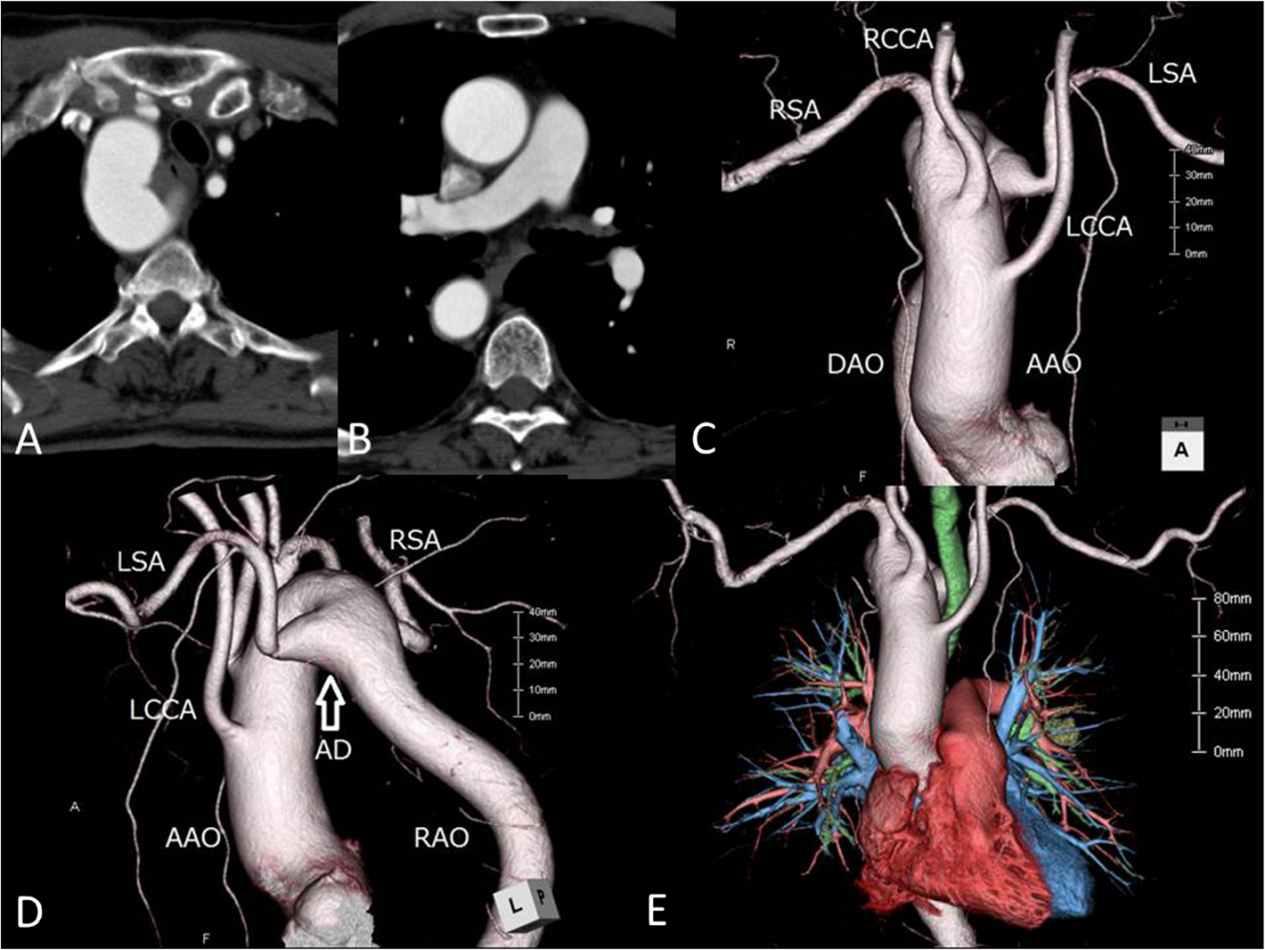
**A chest computed tomography scan. A**) The axial view shows the right aortic arch. **B**) The coronal view shows the right descending aorta in the right thoracic cavity. **C**) The anterior view of the three-dimensional computed tomography scan shows the right aortic arch with the aberrant left subclavian artery. **D**) The lateral view shows the so-called “Kommerell diverticulum” of the left subclavian artery. **E**) The branching of the pulmonary artery, vein, and the bronchi are normal. AAO, ascending aorta; AD, aortic diverticulum; DAO, descending aorta;. LCCA, left common carotid artery; LSA, left subclavian artery; RAO, right aorta; RCCA, right common carotid artery; RSA, right subclavian artery.

A 71-year-old man was referred to our department with a diagnosis of squamous cell carcinoma of the lung, clinical stage IA. Computed tomography showed a pulmonary tumor 2 cm in diameter in the left lower lobe and a right aortic arch (Figure 1, A and B). Three-dimensional chest computed tomography revealed that three arteries originated from the ascending aorta in order of the left common carotid artery, the right common carotid artery, and the right subclavian artery (Figure 1, C). Moreover, it showed that the left subclavian artery branched from the descending aorta and that the origin of the left subclavian artery had dilated into a cystic form, known as a Kommerell diverticulum (Figure 1, D). There were no abnormalities of the pulmonary arteries, veins, or bronchi (Figure 1, E). The lengths of the left and right bronchi were 4.8 and 1.2 cm, respectively. It is normal size for adult male. The anatomy of the brachiocephalic veins and the superior vena cava was normal. They located anterior to the trachea and posterior to the ascending aorta. The azygos vein drained into the superior vena cava as usual. Echocardiography revealed no cardiac abnormalities.

**Figure 2:**
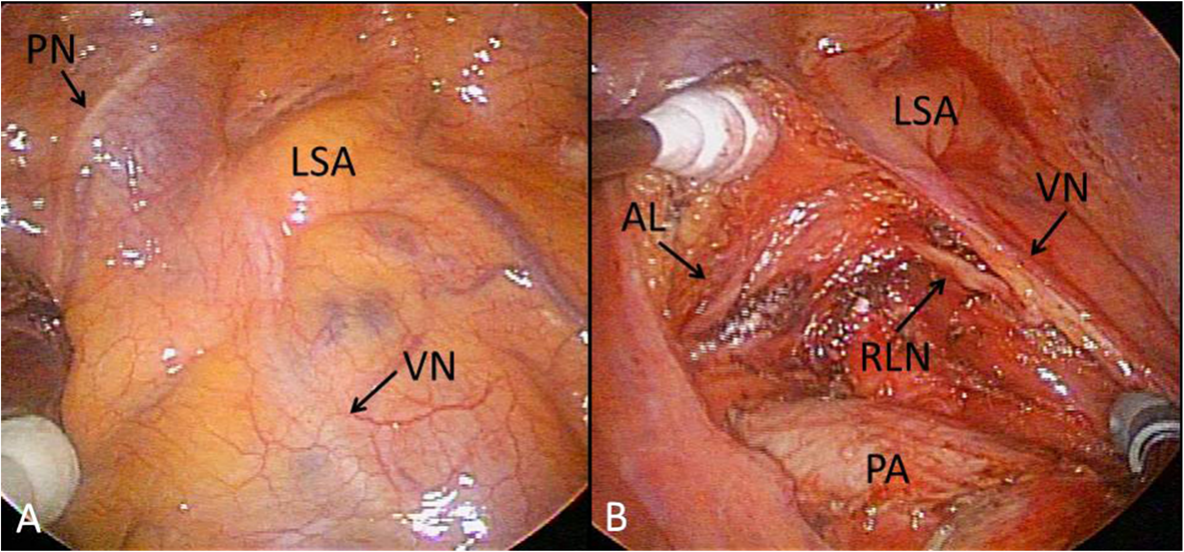
**Intraoperative thoracoscopic view of the left upper mediastinum. A**) An intraoperative view before the lymph node dissection of the left upper mediastinum. There was no aortic arch. The vagus nerve, which passed over the left subclavian artery, was identified. **B**) An intraoperative view after the upper mediastinal lymph node dissection. The recurrent laryngeal nerve, which branched from the vagus nerve, was identified. The recurrent laryngeal nerve passed beneath the arterial ligament. AL, arterial ligament; LSA, left subclavian artery; PA, pulmonary artery; PN, phrenic nerve; RLN, recurrent laryngeal nerve; VN, vagus nerve.

The patient underwent a VATS left lower lobectomy and mediastinal lymph node dissection. All procedures were performed with 3-cm anterior and 2-cm posterior access ports in the 5th and 6th intercostal spaces without rib splitting. There was no aortic arch or descending aorta in the left thoracic cavity, but the esophagus. There was no anomaly in the location and branching of the pulmonary arteries, veins, bronchi, and the lobulation of the lungs. A left lower lobectomy was performed in the usual manner. The upper mediastinal lymph node dissection followed the lower mediastinal dissection. First, we identified the vagus nerve at the level of the left pulmonary artery. When we proceeded with the dissection in the cranial direction, the arterial ligament was found between the left subclavian artery and the left pulmonary artery. It measured 4.3 cm length and 0.4 cm in width. The RLN, which branched from the vagus nerve, was recurrent around the left subclavian artery (Figure 2, A and B). A Kommerell diverticulum was found at the origin of the left subclavian artery. The patient was discharged without any complications such as hoarseness.

## Discussion

A right aortic arch was classified into three types by Stewart et al [1]. Type I, which is a mirror image of the normal anatomical structure, accounts for 59% of cases. This type is often accompanied by visceral inversion. Of type I cases, 98% have congenital heart disease such as tetralogy of Fallot, truncus arteriosus communis, and ventricular septal defect. Type II accounts for 40% of cases, including our case. This type has an aberrant left subclavian artery that originates from the descending aorta. The stem of the left subclavian artery is usually dilated and called a Kommerell diverticulum. Of type II cases, 12% have congenital heart disease. Type III, which involves an isolated left subclavian artery, comprises only about 1% of cases [1,2].

There have only been seven cases with a right aortic arch, including our case, in which the patient underwent pulmonary resection for left lung cancer. According to the classification of Stewart and colleagues, one of these cases was a type I right aortic arch, and six were type II. Five surgeries were performed through an open thoracotomy and two were performed under VATS. Six patients underwent upper mediastinal lymph node dissection following the lobectomy. In all six cases, the RLN was identified. The RLN was recurrent around the arterial ligament in five cases and around the left subclavian artery in one case. We performed a lobectomy without any difficulty because there was no abnormality in location and branching of the pulmonary arteries, veins, and the bronchi [3]. In the case of left lung cancer, there have been several reports in which mediastinal lymph node dissection was easily performed, compared with right lung cancer, because there was no aortic arch in the left thoracic cavity [4]. However, it was difficult to decide the range of lymphadenectomy and to number the stations of lymph nodes [2]. We interpreted lymph nodes around the arterial ligament as lower paratracheal lymph nodes (#4) [5]. Because the lymphatic drainage from the left lung to those areas goes along the tracheobronchus, we thought they should be classified as paratracheal lymph nodes. According to a certain report, lymph nodes around the arterial ligament were interpreted as subaortic lymph nodes (#5) and lymph nodes around the left subclavian artery were regarded as para-aortic lymph nodes (#6) [2].

We believe that this is the first case report in which complete thoracoscopic surgery without a mini-thoracotomy was performed for lung cancer with a right aortic arch. Because we verified the location and branching of the vessels by preoperative three-dimensional computed tomography and understood the anatomy, we could anticipate the site of the RLN. Furthermore, a magnified view by thoracoscopy enabled us to identify the vagus nerve, RLN, and arterial ligament easily and to perform the mediastinal lymph node dissection safely.

## Conclusion

Although an advanced technique is required for thoracoscopic surgery, it is feasible to perform VATS lobectomy on patients with a right aortic arch.

## Consent

Written informed consent was obtained from the patient for publication of this report and any accompying images.

## Competing interests

The authors declare that they have no competing interests.

## Authors’ contributions

All the authors participated in the treatment of this patient. H. Wada drafted the manuscript. Y. Hida, K. Kaga, R. Hase, K. Ohtaka, J. Muto, R. Nakada-Kubota, S. Hirano and Y. Matsui helped to draft the manuscript. Y. Hida revised it critically. H. Wada provided the figures. Y. Hida performed the operation. All authors read and approved the final manuscript.
